# Hypertrophic Osteopathy Associated With Intrathoracic Masses in 5 Dogs and Review of the Literature

**DOI:** 10.1155/vmi/1441615

**Published:** 2026-02-17

**Authors:** Mehmet Alper Cetinkaya, Soner Cagatay, Mehmet Pilli, Ali Curukoglu, Deniz Seyrek Intas

**Affiliations:** ^1^ Laboratory Animal Research and Application Centre, Hacettepe University, Sihhiye, Ankara, Türkiye, hacettepe.edu.tr; ^2^ Department of Surgery, Faculty of Veterinary Medicine, Near East University, Nicosia, Cyprus, neu.edu.tr

**Keywords:** dog, hypertrophic osteoarthropathy, hypertrophic osteopathy, review, thoracic mass

## Abstract

This study describes the clinical and radiographical findings of hypertrophic osteopathy in 5 female mature adult dogs with pulmonary and mediastinal masses. Besides, the literature review from the past to today provides information about the disease.

## 1. Introduction

Hypertrophic osteopathy (HO) is a rare disease condition, usually secondary to a primary thoracic mass (neoplasm and infection) or, less commonly, cardiovascular system abnormalities or abdominal mass (neoplasm and infection). In reaction to the existence of a mass(es), nonedematous soft tissue swellings and diffuse periosteal new bone formation develop in all distal extremities, causing mild to severe lameness. The prognosis and treatment depend highly on the primary disease [1–4].

## 2. Case Descriptions

Five dogs presented with lameness, swelling on the distal portions of the extremities, and reluctance to walk, which comprised the cases for this study. All dogs were crossbred, female (four neutered), and over 8 years of age. Before the procedures, the patients’ owners were informed, and their signed consent was requested.

## 3. Clinical and Radiographical Findings and Discussion

**FIGURE 1:**
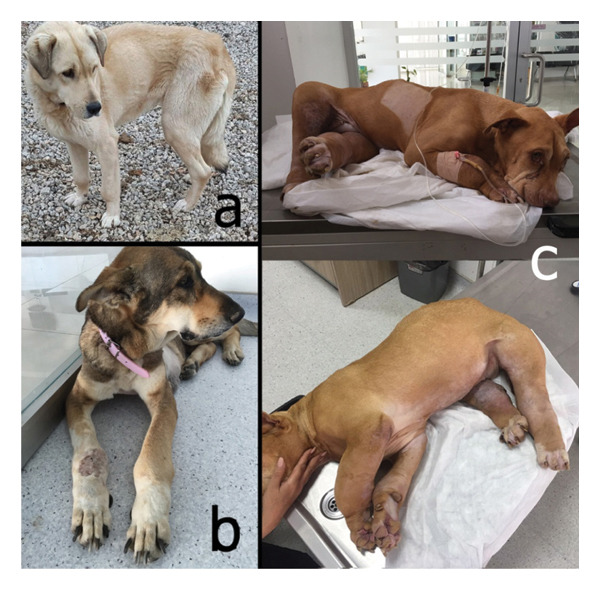
Clinical appearance of some cases. Severity of the HO is mild in Case 3 (a), moderate in Case 5 (b), and severe in Case 4 (c).

Clinical examination revealed mild to severe nonedematous swelling of the distal parts of all extremities (Figures 1(a), 1(b), and 1(c)); this was more severe and included the proximal parts of the four limbs in the fourth case (Figure 1(b)). In Case 5, a subcutaneous lipoma was also detected in the sternal region.

**FIGURE 2:**
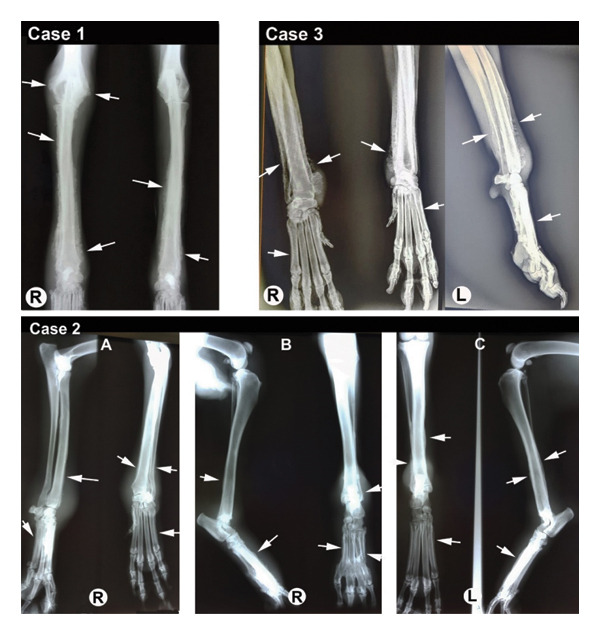
Radiographical views of extremities in cases 1 to 3; (case 1) CrCa view of the right and left distal humerus, RU and carpal joints reveals a palisading appearance of bilateral proliferative periosteal reactions extending distally to proximally in RU and metacarpal bones, and limited only distal humerus (white arrows); the right side is affected more severely; (case 2) mediolateral and CrCa views of the right antebrachium (A), mediolateral and CrCa views of the right tibia‐fibula (B), and CrCa and mediolateral views of the left tibia‐fibula (C) show a palisading appearance of a mild proliferative periosteal reaction of distal RU and metacarpal bones (white arrows). On the tibia, the irregular periosteal reaction is more distinguishable on the left side than the right side, and the entire periosteal surface of the metatarsal bones is affected (white arrows). Soft tissue swelling is also apparent on the distal portions of the extremities; (case 3) CrCa view of the right and left, and the mediolateral view of the left carpal joints show an irregular bilateral proliferative reaction of RU and metacarpal bones (white arrows) limited on the distal side.

**FIGURE 3:**
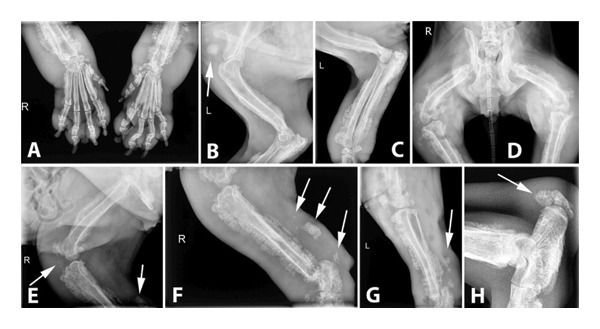
Radiographical views of extremities in Case 4. HO lesions also appeared as mineralization in soft tissues (white arrows). Periosteal reactions cover all affected bones from distal to proximal, and all lesions are bilateral. Extensive soft tissue swelling is also evident even in the proximal parts of the extremities (Figure 1(c)). (A) CrCa views of right and left distal antebrachiums and metacarpal and phalangeal bones. (B) Mediolateral view of the left shoulder, humerus, and the cubiti. (C) Mediolateral view of the left humerus, cubiti, antebrachium, and carpal joint. (D) Dorsoventral view of the pelvis, hip joints, femurs, stifle joints, and proximal tibias. (E) Mediolateral view of the right hip joint, femur, stifle joint, and proximal tibia. (F) Mediolateral view of the right stifle joint, tibia, and the tarsal joint. (G) Mediolateral view of the left stifle joint and tibia. (H) Mediolateral view of the left calcaneus. Note a massive enthesophyte formation on the calcaneus (white arrow).

**FIGURE 4:**
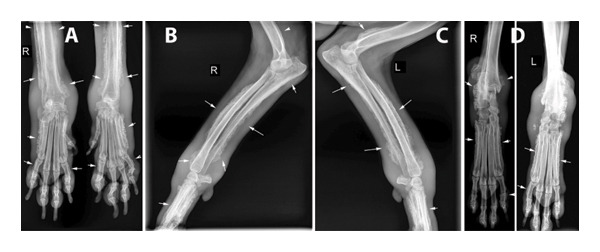
Radiographical views of extremities in Case 5. (A) CrCa views of right and left distal antebrachium and metacarpal and phalangeal bones. (B) Mediolateral view of the right distal humerus, cubiti, antebrachium, and metacarpus. (C) Mediolateral view of the left distal humerus, cubiti, antebrachium, and metacarpus. (D) CrCa views of right and left distal tibias, tarsal joints, and metatarsus. Note the irregular surfaced, periosteal proliferative lesions on the affected bones (white arrows) and soft tissue swellings on the extremities (case in Figure 1(b)).

Radiographs of the extremities revealed bilateral symmetrical periosteal, palisading new bone formation consistent with HO in all cases (Figures 2, 3, and 4). Soft tissue swelling was also evident on radiographs in some cases Figures 2 (case 2), 3, and 4). Affected bones were scapula (Case 4), humerus (Cases 1, 4, and 5), radius–ulna (Cases 1 to 5), pelvis (Case 4), femur (Case 4), tibia–fibula (Cases 2–5), calcaneus (Cases 4 and 5), patella (Case 4), metacarpus/‐tarsus (Cases 1 to 5), and phalanges (Cases 3, 4, and 5), and all these lesions were bilateral. In addition, mineralization foci were observed in the synovial membranes of joint capsules, tendovaginas, and bursal sacs in Case 4 (Figure 3); this case was unique because we did not encounter any report in veterinary medicine about soft tissue mineralization in HO cases.

**FIGURE 5:**
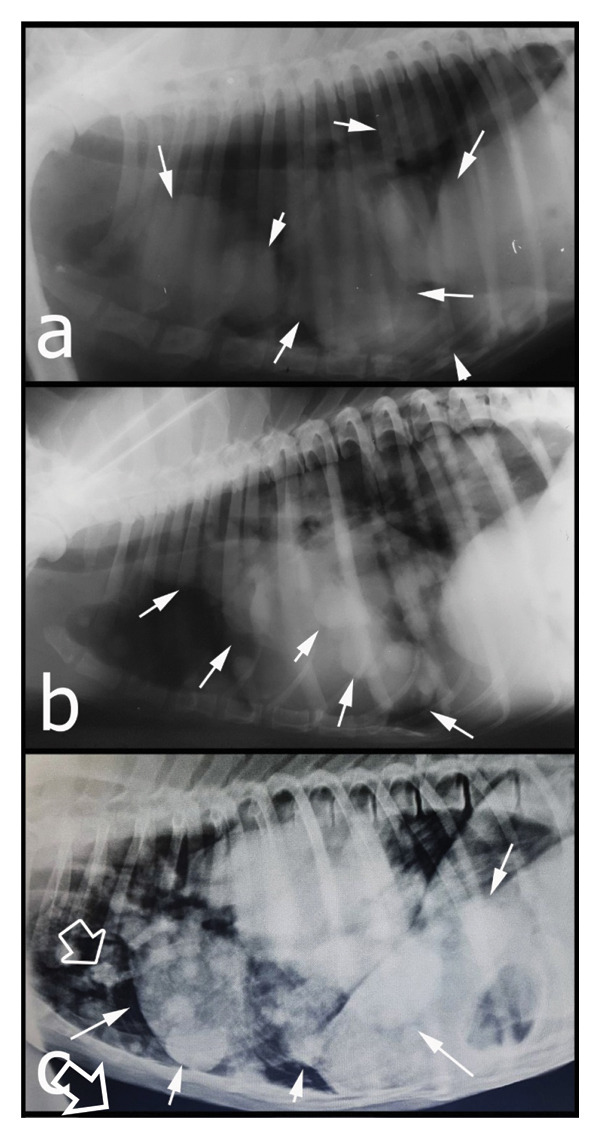
Thoracic radiographs of Case 1 (a), Case 2 (b), and Case 3 (c) show multiple pulmonary masses (white arrows indicate some of them). These masses vary in size and are distributed throughout the entire lung field. In Case 3, there is also evidence of mild mineralization within the mass (open arrow).

**FIGURE 6:**
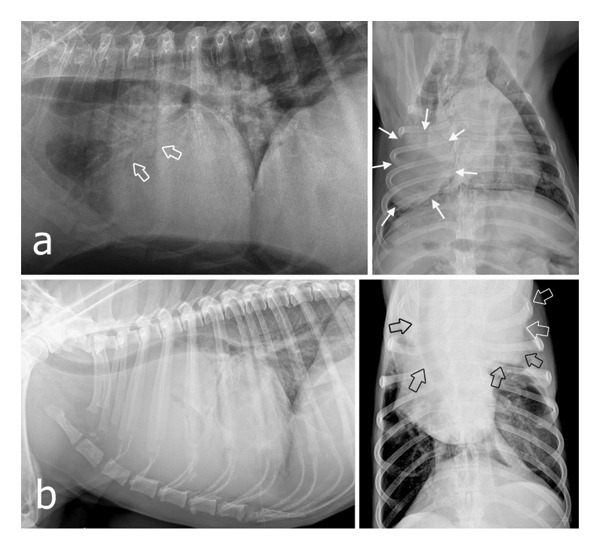
Lateral and dorsoventral/ventrodorsal radiographic views of the thorax in Case 4 (a) and Case 5 (b) reveal single thoracic masses (white arrows). Case 4 shows a mixed lung pattern with air bronchograms (open arrows), most likely edema due to pressure by the mass. Case 5 demonstrates a huge mediastinal mass superimposing the cranial lung field and silhouetting with the heart. The mass displaces the trachea dorsally and to the right, as well as the heart (open arrows). The left caudodorsal lung field also shows a diffuse interstitial lung pattern. There is a mild alveolar pattern in the hilar region.

Abdominal radiographs of all cases and abdominal ultrasonographic examination of Cases 4 and 5 revealed no abnormal abdominal findings, whereas thorax radiographs revealed pulmonary and mediastinal masses (Figures 5 and 6). Three were observed as multiple masses, and two were observed as a single mass. In the 4th and 5th cases, whose owners allowed ultrasound‐guided fine‐needle aspiration biopsies of the pulmonary masses, cytological examination confirmed carcinoma; however, tumor subtyping was not possible based on cytology alone. In Case 4, despite the presence of soft tissue mineralization on imaging, no cytological features allowed further classification.

Since the prognosis in dogs with secondary HO due to pulmonary masses is quite poor, in all patients, euthanasia was recommended instead of any surgery. In Cases 2 and 4, the owners accepted euthanasia, but in Cases 1, 3, and 5, the owners did not approve of any other option than palliative care. Nonsteroidal anti‐inflammatory drugs were recommended for these patients. According to information obtained by phone calls, these patients died within two months.

## 4. Review of Literature

HO is an uncommon bone disorder secondary to a primary disease. It commonly occurs due to neoplastic or infectious masses in the thoracic cavity or, less often, in the abdominal cavity [1–4]. Additionally, only in humans, rare cases of a primary hereditary form of hypertrophic osteoarthropathy (HOA) exist, and the genetic background has recently been discovered [5–9].

Different terminologies exist for HO, including hypertrophic pulmonary osteopathy, HOA, hypertrophic pulmonary osteoarthropathy, pulmonary osteoarthropathy, osteoporosis deformans, achropachia, Marie’s disease, and Marie–Bamberger syndrome [3, 10].

Archaeological findings have confirmed that HO also existed in ancient times, both in humans and animals [11, 12]. Although Hippocrates first described digital clubbing (swelling of tissue with loss of normal angle between nail and nail bed) as a sign of severe lung disease almost 2500 years ago, the disease was first observed in modern times by Bamberger in 1889 and Pierre Marie in 1890; therefore, this pathology is named as Marie–Bamberger syndrome [13, 14].

In humans, the term HOA is preferred to describe this disorder because it is characterized by digital clubbing, arthralgia/arthritis, and increased periostal activity of the tubular bones [8, 9, 15]. Unlike in humans, the term HOA is not used in veterinary medicine because in animals, joints are not directly affected in this disease. However, periarticular soft tissue swelling may cause a decrease in joint range of motion [1–4]. HO is most commonly observed in dogs [4, 16–26] but was also reported in other animal species [27–41].

In humans, HOA can also occur as a primary disease called pachydermoperiostosis; this form is generally familial and autosomal‐dominant or autosomal‐recessive. Pachydermia, as skin thickening and excessive sweating, will be observed with or without characteristic symptoms of HOA [6–9, 42]. Congenital heart disease, especially patent *ductus arteriosus*, was also observed in patients with primary HOA [43, 44]. Pachydermoperiostosis usually begins soon after puberty, progresses for five to ten years, and remains unchanged throughout life. The incidence of the syndrome is significantly higher in males (ratio of 7–9:1), and males also tend to experience more severe symptoms than females [45, 46]. Another primary type of HOA, named cranio‐osteoarthropathy, appears as delayed closure of the cranial sutures in humans without pachydermia [7, 47, 48].

HO only appears as a secondary abnormality resulting from intrathoracic or intra‐abdominal masses in animals. Metastatic pulmonary neoplasms [1, 19, 27, 28], pulmonary infections [34, 39, 49, 50], bronchial foreign bodies [49, 51], *Spirocerca lupi* granulomas [16, 25], *Dirofilaria immitis* [16], nodular lung lesions caused by *Paragonimus westermani* [26], congenital megaesophagus [52], embryonal rhabdomyosarcoma of the esophagus [53], canine tuberculosis [20], aortic valvular endocarditis [18], infective endocarditis [16, 21], and ventricular septal defect [54] are examples of intrathoracic lesions related to the disease. Intra‐abdominal lesions associated with the disease include botryoid rhabdomyosarcoma of the urinary bladder [17], prostatic adenocarcinoma [55, 56], ovarian neoplasia [31], liver adenocarcinoma [23, 57], renal neoplasia [22], adrenocortical carcinoma [58], and *Cryptococcus neoformans* and *Hepatozoon* sp. infection [37, 59]. Other reported diseases include systemic granulomatous disease [35] and injection‐site sarcoma without evidence of intrathoracic or intra‐abdominal involvement [36].

Because of the primary lesion, bilateral symmetrical, nonedematous soft tissue swelling occurs on the distal portions of all extremities and generally starts on the phalanges and moves proximally with time. Following these, diffuse palisading periosteal new bone formation without damaging the cortical bone appears on the outer cortex of the diaphyses. These are not primary neoplasia or metastatic bone lesions and cause mild to severe lameness; severely affected animals will be reluctant to walk and will barely be able to stand. Periosteal new bone formation is most commonly perpendicular to the cortex with an irregular palisading pattern, whereas a smooth periosteal reaction parallel to the cortex represents organized or remodeled lamellar bone and is typically associated with a chronic process [1–5]. As nonedematous soft tissue swelling moves proximally, periosteal proliferations will also spread proximally to involve the humerus and scapula, femur, and pelvis; mandibles, ribs, and vertebrae may also be affected [1–5, 32, 60]. Inflammation of the synovial membrane and swollen, painful joints have only been reported in humans [8, 9, 15]. Although joints are not affected in animals, the joint range of motion might be limited because of excessive periarticular soft tissue swelling [3–5].

Researchers have suggested various theories regarding the possible disease mechanism and the associated periosteal reaction, even though the pathogenesis itself has not yet been fully understood. Neurovascular reflex theory and platelet clumps/megakaryocyte theory are the two most widely accepted theories [1].

The autonomic neurovascular reflex originates from the thoracic region and is carried by afferent vagal fibers. This reflex leaves the lung near the bronchi and joins the vagal nerve in the mediastinum. Furthermore, an alternative afferent pathway might originate from the parietal pleura and travel along the intercostal nerves. It is also thought that extrapulmonary lesions follow the distribution of the vagopharyngeal and vagus nerves, which contain fibers that innervate vascular tissues. The vagus and intercostal nerves likely carry afferent impulses along the pathway from the lesion toward the central nervous system, and these impulses are thought to initiate the HO. This reflex enhances circulation in the distal extremities; additionally, connective tissue and the periosteum are also affected [3, 4, 61]. The improvement in symptoms following vagotomy or intercostal nerve resection supports this theory [28, 61, 62]. In veterinary medicine, the neurovascular reflex theory has gained broad acceptance.

More recently, the megakaryocyte–platelet clump theory has been proposed to explain the pathogenesis of HO [63]. Originating primarily in the bone marrow, megakaryocytes are giant cells responsible for producing platelets. These large cells enter the bloodstream, where their massive size causes them to become trapped in the pulmonary capillaries; subsequently, they generate mature platelets through a budding process. Megakaryocytes are found in significant numbers within the lungs and pulmonary circulation, where both they and platelet clumps normally undergo fragmentation. However, in abnormal conditions such as cardiovascular shunts, pulmonary arteriovenous shunts, or pulmonary tumors that cause vascular shunts, megakaryocytes and platelet clumps fail to undergo normal fragmentation, and they potentially can enter the systemic circulation, deposit in the distal vasculature, and consequently release platelet‐derived growth factor (PDGF) [64]. The localized release of these growth factors stimulates fibroblast proliferation and increases both vascularity and permeability, ultimately leading to connective tissue alterations and a periosteal reaction [65]. Another factor potentially contributing to pathogenesis is vascular endothelial growth factor (VEGF). Like PDGF, VEGF is also platelet‐derived. Furthermore, VEGF is a potent stimulator of new blood vessel growth (angiogenesis) and osteoblast development, and its production increases under hypoxic and malignant circumstances [14]. Elevated VEGF levels have been reported in both primary and secondary forms of HOA associated with lung cancer [66]. However, the megakaryocyte–platelet clump theory fails to explain clinical improvement after vagotomy in some cases.

Based on current reports, HO does not appear to have an intrinsic breed or sex predisposition. As HO is a secondary condition, demographic factors such as breed, sex, and age largely reflect those of the underlying primary disease rather than HO itself. Consequently, HO is most commonly reported in adult animals due to its frequent association with neoplasia. Apparent sex‐related differences in HO prevalence are therefore primary disease dependent. For example, a higher frequency in females has been reported in cases associated with mammary carcinomas, as pulmonary metastases from these tumors may trigger HO [2]. In contrast, other underlying conditions may show different sex distributions. Thus, any observed sex predilection for HO should be interpreted in relation to the primary disease and species involved, rather than as a characteristic of HO per se.

The prognosis greatly depends on the underlying cause of HO. The management of HOA in humans is classified as either treatment of the underlying etiology or symptomatic therapy. Treatment of the primary cause includes tumor resection, radiotherapy, chemotherapy, treatment of infection, replacement of infected grafts, organ transplantation, and surgical correction of cyanotic heart disease. Symptomatic treatments include vagotomy, bisphosphonates, octreotide, application of epidermal growth factor receptor (EGFR) inhibitor, and adrenergic antagonists [19, 63].

The ideal treatment for HO in dogs depends on the underlying cause. Treating the underlying health problem could lead to the regression of bone abnormalities. Treatments for HO include resection of the tumor (intrathoracic or intra‐abdominal), chemotherapy, vagotomy, treatment of infection, surgically treating heartworm disease (dirofilariasis), and treating spirocercal granulomas. For cases of HO caused by heartworm infestation or primary lung tumors, surgery is a viable treatment option [3, 19, 32, 58, 62].

Nonsteroidal anti‐inflammatory drugs will be suitable for providing palliative care to dogs. Dogs suffering from HO due to secondary pulmonary cancer have an abysmal prognosis, and euthanasia is normally preferred instead of surgery [2].

## Funding

This research received no grant from any funding agency/sector.

## Disclosure

This study was carried out in the Near East University Faculty of Veterinary Medicine, Animal Hospital.

## Conflicts of Interest

The authors declare no conflicts of interest.

## Data Availability

The data that support the findings of this study are available from the corresponding author upon reasonable request.
